# Anti-mitochondrial autoantibodies are associated with cardiomyopathy, dysphagia, and features of more severe disease in adult-onset myositis

**DOI:** 10.1007/s10067-021-05730-7

**Published:** 2021-04-13

**Authors:** Sara E. Sabbagh, Iago Pinal-Fernandez, Maria Casal-Dominguez, Jemima Albayda, Julie J. Paik, Frederick W. Miller, Lisa G. Rider, Andrew L. Mammen, Lisa Christopher-Stine, for the Johns Hopkins Myositis Center Group, Harlan Michelle, Eleni Tiniakou, Sonye K. Danoff, Tom Lloyd

**Affiliations:** 1 Muscle Disease Unit, Laboratory of Muscle Stem Cells and Gene Regulation, National Institute of Arthritis and Musculoskeletal and Skin Diseases, National Institutes of Health, Bethesda, MD USA; 2 Division of Rheumatology, Department of Pediatrics, Medical College of Wisconsin, Milwaukee, WI USA; 3 Department of Neurology, Johns Hopkins University School of Medicine, Baltimore, MD USA; 4 Faculty of Health Sciences, Universitat Oberta de Catalunya, Barcelona, Spain; 5 Department of Medicine, Johns Hopkins University School of Medicine, Baltimore, MD USA; 6 Environmental Autoimmunity Group, National Institute of Environmental Health Sciences, National Institutes of Health, Bethesda, MD USA; 7 Johns Hopkins Myositis Center, Division of Rheumatology, Johns Hopkins University School of Medicine, Bayview Medical Office, 5200 Eastern Ave #301, Baltimore, MD 21224 USA

**Keywords:** Anti-mitochondrial autoantibodies, Juvenile myositis, Myositis

## Abstract

We analyzed the prevalence of anti-mitochondrial autoantibodies (AMA) in adult- and juvenile-onset myositis longitudinal cohorts and investigated phenotypic differences in myositis patients with AMA. We screened sera from myositis patients including 619 adult- and 371 juvenile-onset dermatomyositis (DM, JDM), polymyositis (PM, JPM), inclusion body myositis (IBM), or amyopathic DM patients and from healthy controls, including 164 adults and 92 children, for AMA by ELISA. Clinical characteristics were compared between myositis patients with and without AMA. AMA were present in 5% of adult myositis patients (16 of 216 DM, 10 of 222 PM, 4 of 140 IBM, 1 of 19 amyopathic DM), 1% of juvenile myositis patients (3 of 302 JDM, 1 of 25 JPM), and 1% of both adult and juvenile healthy controls. In patients with adult-onset myositis, AMA were associated with persistent muscle weakness, Raynaud’s phenomenon, dysphagia, and cardiomyopathy. Adult myositis patients with AMA may have more severe or treatment refractory disease, as they more frequently received glucocorticoids and intravenous immunoglobulin. In juvenile myositis, children with AMA often had falling episodes and dysphagia, but no other clinical features or medications were significantly associated with AMA. AMA are present in 5% of adult myositis patients and associated with cardiomyopathy, dysphagia, and other signs of severe disease. The prevalence of AMA is not increased in patients with juvenile myositis compared to age-matched healthy controls. Our data suggest that the presence of AMA in adult myositis patients should prompt screening for cardiac and swallowing involvement.

**Table:** 

**Key Points** *• Approximately 5% of a large North American cohort of adult myositis patients have anti-mitochondrial autoantibodies.* *• Adults with anti-mitochondrial autoantibodies often have chronic weakness, Raynaud’s, dysphagia, cardiomyopathy, and more severe disease.* *• Anti-mitochondrial autoantibodies are rare in juvenile myositis and not associated with a specific clinical phenotype.*

**Supplementary Information:**

The online version contains supplementary material available at 10.1007/s10067-021-05730-7.

## Introduction

Idiopathic inflammatory myopathies (IIM), including dermatomyositis (DM) and polymyositis (PM), are a heterogeneous group of systemic autoimmune diseases characterized by inflammation of skeletal muscles, rashes, and autoantibodies which define clinical subgroups [1]. Anti-mitochondrial autoantibodies (AMA) are typically found in association with primary biliary cirrhosis (PBC) but have also been observed in other autoimmune diseases [2]. Previously, we described 7 AMA-positive patients who often had cardiac involvement, muscle atrophy, and a chronic disease course [3]. However, these clinical associations with AMA have been questioned by others [4]. In juvenile-IIM (JIIM), the prevalence and significance of AMA are unknown. Overall, associations of AMA in myositis are poorly defined, and descriptions of AMA are limited. In this study, we systematically analyzed the prevalence of AMA in both adult and JIIM longitudinal cohorts and investigated phenotypic differences between adult and JIIM patients with or without AMA.

## Patients and methods

### Patients and serum samples

Serum samples stored at −80°C from 619 adults sequentially enrolled in the Johns Hopkins IRB-approved Myositis Center Longitudinal Cohort study between 2011 and 2015 and from 164 healthy control adults enrolled at National Institutes of Health (NIH) IRB-approved studies were available. Patients were classified as DM, PM, inclusion body myositis (IBM), or amyopathic DM based on Bohan and Peter [5], Griggs [6], or Sontheimer criteria. Screening for myositis-specific autoantibodies (MSAs) and evaluation of strength, rashes, dysphagia, and interstitial lung disease (ILD) were completed as previously described [7]. Retrospective medical record review was completed for 619 patients regarding cardiac involvement, defined as cardiomyopathy, heart block, atrial tachycardia, and/or myocarditis [3]. Thirty-one patients had cardiomyopathy, of whom 16 had ECHOs, 5 had cardiac MRI and ECHOs, 3 had cardiac biopsy with ECHO and/or cardiac MRI, and 7 had physician documentation of cardiomyopathy; however, prior diagnostic records were not available. EKGs were available in 24/30 patients with heart block and/or atrial tachycardia. One patient had myocarditis, diagnosed by the combination of EKG, ECHO, and cardiac MRI. Of the 7 AMA-positive patients in our original case series [3], 3 patients were not seen between 2011 and 2015 and thus not included in our current study. Of the remaining 4 patients, 2 had cardiomyopathy and 2 had no cardiac involvement. Of our cohort of 619 sequential patients, 480 MSA-positive or IBM patients had complete clinical data available for further analysis.

Patients from the Childhood Myositis Heterogeneity Collaborative Study [8] enrolled in NIH IRB-approved studies between 1989 and 2016, consisting of 371 patients with probable or definite JIIM [5] and 92 healthy control children, were included. A physician questionnaire captured demographics, clinical and laboratory features, as well as therapeutic usage and responses [8, 9].

### Anti-mitochondrial autoantibody detection

An enhanced performance AMA ELISA [M2 EP (MIT3), Quanta Lite, INOVA Diagnostics, San Diego, CA] was performed according to the manufacturer’s instructions. The reactivity for each sample was calculated by dividing the sample OD by the low positive OD and multiplying by 25, then classified as negative (<25) or positive (≥25).

### Statistical analysis

Dichotomous variables were expressed as percentages and absolute frequencies, and continuous variables were reported as means and standard deviations. Pairwise comparisons for categorical variables were made using *χ*^2^ test or Fisher’s exact test; continuous variables were compared using Student *t*-test. Multivariate comparisons were performed using linear regression for continuous variables and logistic regression for dichotomous variables. All multivariate comparisons were adjusted by gender and clinical (IBM) or autoantibody group. Multivariate comparisons were also separately adjusted for anti-Ro52 autoantibodies. All statistical analyses were performed using Stata/MP V.14.1. A two-sided *p* value of ≤0.05 was considered statistically significant.

## Results

### Prevalence and demographics of adult and juvenile patients with AMA

AMA were present in 32 of 619 (5%) adult myositis patients and in 1 of 164 (0.6%) adult healthy controls (*p*=0.004). AMA were also present in 4 of 371 (1%) JIIM patients and 1 of 92 (1%) juvenile healthy controls. Of the 480 IIM patients with complete clinical data available, 30 (6%) patients had AMA, 5 of whom had PBC, and the majority of whom were female (80%) (Table 1). Although there was no difference in the distribution of MSAs, patients with AMA more frequently had anti-Ro52 autoantibodies (47% vs 29%) (Table 1).

**Table 1:** General features of adult myositis patients with and without AMA

	AMA-positive (*N*=30) % (n/N) or mean (SD)	AMA-negative (*N*=450) % (n/N) or mean (SD)	Univariate *p*-value	Multivariate *p*-value	Total (*N*=480)
Female sex	80% (24)	61% (276)	0.04		62% (300)
Race					
White	67% (20)	75% (337)	0.3	0.2	74% (357)
Black	17% (5)	18% (82)	0.8	0.9	18% (87)
Other races^a^	17% (5)^b^	7% (31)	0.06	0.05	8% (36)
Age of onset (years)	49.4 (14.9)	52.9 (15.4)	0.2	0.6	52.7 (15.4)
Follow-up time (years)	3.4 (2.8)	3.5 (3.4)	0.9	0.4	3.4 (3.3)
Myositis autoantibody groups					
Anti-TIF1γ	23% (7)	10% (47)	0.06		11% (54)
Anti-NXP2	17% (5)	8% (38)	0.2		9% (43)
Anti-MDA5	10% (3)	5% (22)	0.2		5% (25)
Anti-Mi-2	10% (3)	7% (31)	0.5		7% (34)
Anti-SRP	3% (1)	6% (27)	1.0		6% (28)
Anti-HMGCR	10% (3)	13% (60)	0.8		13% (63)
Anti-PL-12	3% (1)	2% (11)	0.5		2% (12)
Anti-PL-7	0% (0)	2% (10)	1.0		2% (10)
Anti-Jo-1	7% (2)	13% (58)	0.6		12% (60)
Anti-Ro52	47% (14)	29% (131)	0.04	0.01	30% (145)
Myositis clinical groups					
IBM	17% (5)	32% (146)	0.07		31% (151)

Dichotomous variables were expressed as percentage (count) and continuous variables as mean (SD). Univariate comparisons of continuous variables were made using Student’s *t*-test, while dichotomous variables were compared either using chi-squared test or Fisher’s exact test, as appropriate. Multivariate comparisons were performed using linear regression for continuous variables and logistic regression for dichotomous variables. All multivariate comparisons were adjusted by gender and clinical group (inclusion body myositis or autoantibody group)

^a^Non-Caucasian, non-African American, or unknown; ^b^unknown: 3, Asian: 2

*AMA* anti-mitochondrial autoantibodies, *TIF1* transcription intermediary factor 1, *NXP2* nuclear matrix protein-2, *MDA5* melanoma differentiation associated protein-5, *SRP* signal recognition particle, *HMGCR* 3-hydroxy-3-methylglutaryl-CoA reductase, *IBM* inclusion body myositis

### Clinical features and medications received among patients with AMA

Adult myositis patients with AMA did not have more muscle weakness at disease onset but had a higher prevalence of weakness throughout disease course (90% vs 62%) and more often had dysphagia (63% vs 36%) (Table 2). AMA-positive patients also more often had Gottron’s papules and/or heliotrope rash (60% vs 41%) and Raynaud’s phenomenon (43% vs 14%), despite similar frequency of DM in patients with and without AMA. Patients with AMA also more often had cardiomyopathy (16% vs 5%). However, the type of cardiomyopathy was variable (dilated: 1, non-ischemic: 2, unspecified: 2), and there was no difference in the presence of other cardiac manifestations (Table 2). Adult myositis patients with AMA more often received corticosteroids (90% vs 67%) and intravenous immunoglobulin (60% vs 29%) and overall received a higher number of medications (Table 2). Notably, muscle biopsies of AMA patients did not have increased mitochondrial dysfunction [10] (Supplemental Table 1).

**Table 2:** Clinical features and medications received in adult myositis patients with and without AMA

	AMA-positive % (n/N) or mean (SD)	AMA-negative % (n/N) or mean (SD)	Univariate *p*-value	Multivariate *p*-value	Total
	(*N*=32)	(*N*=587)			(*N*=619)
Heart involvement					
Heart involvement^a^	16% (5/32)	8% (49/587)	0.2	0.2	9% (54/619)
Cardiomyopathy	16% (5/32)	5% (26/587)	0.02	0.01	5% (31/619)
	(*N*=30)	(*N*=450)			(*N*=480)
Muscle weakness					
At disease onset	43% (13/30)	42% (191/450)	0.9	0.9	42% (204/480)
At follow-up visits	90% (27/30)	62% (280/450)	0.002	0.001	64% (307/480)
Skin involvement					
DM-specific skin involvement^b^	60% (18/30)	41% (186/450)	0.05	0.4	42% (204/480)
Raynaud’s phenomenon	43% (13/30)	14% (64/450)	< 0.001	< 0.001	16% (77/480)
Mechanics hands	20% (6/30)	20% (92/450)	1.0	0.4	20% (98/480)
Calcinosis	10% (3/30)	9% (42/450)	0.8	0.4	9% (45/480)
Subcutaneous edema	7% (2/30)	13% (60/450)	0.4	0.09	13% (62/480)
Lung involvement					
Interstitial lung disease	20% (6/30)	20% (91/450)	1.0	0.6	20% (97/480)
Esophageal involvement					
Gastroesophageal reflux disease	27% (8/30)	19% (86/450)	0.3	0.7	20% (94/480)
Dysphagia	63% (19/30)	36% (160/450)	0.002	0.01	37% (179/480)
Joint involvement					
Arthritis	20% (6/30)	18% (80/450)	0.8	0.9	18% (86/480)
Arthralgia	47% (14/30)	34% (151/450)	0.1	0.5	34% (165/480)
Systemic involvement					
Fever	10% (3/30)	11% (50/450)	1.0	0.6	11% (53/480)
Cancer-associated myositis	17% (5/30)	11% (49/450)	0.4	0.1	12% (54/480)
Treatments received					
Corticosteroids	90% (27/30)	67% (301/450)	0.008	0.05	68% (328/480)
Azathioprine	30% (9/30)	24% (110/450)	0.5	0.5	25% (119/480)
Methotrexate	40% (12/30)	39% (176/450)	0.9	0.5	39% (188/480)
Mycophenolate	33% (10/30)	22% (99/450)	0.2	0.5	23% (109/480)
IVIG	60% (18/30)	29% (132/450)	< 0.001	0.02	31% (150/480)
Rituximab	27% (8/30)	14% (63/450)	0.07	0.07	15% (71/480)
Total number of medications ^c^	2.8 (1.4)	2.0 (1.5)	0.003	0.009	2.0 (1.5)

Dichotomous variables were expressed as percentage (count) and continuous variables as mean (SD). Univariate comparisons of continuous variables were made using Student’s *t*-test, while dichotomous variables were compared either using chi-squared test or Fisher’s exact test, as appropriate. Multivariate comparisons were performed using linear regression for continuous variables and logistic regression for dichotomous variables. All multivariate comparisons were adjusted by gender and clinical group (inclusion body myositis or autoantibody group)

*IVIG* intravenous immunoglobulin

^a^Myocarditis, atrial tachycardia, heart block, and/or cardiomyopathy

^b^Gottron’s papules and/or heliotrope rash

^c^Total number of medications received at follow-up, including corticosteroids, mycophenolate, methotrexate, IVIG, azathioprine, rituximab, and cyclophosphamide

Dysphagia was seen in slightly higher rates in patients positive for both AMA and anti-Ro52 autoantibodies and those positive for AMA and anti-HMGCR autoantibodies compared to patients who were anti-Ro52-positive/AMA-negative or anti-HMGCR-positive/AMA-negative, respectively (Supplemental Table 2). In addition, patients who were positive for both anti-Ro52 and AMA and those who were positive for anti-synthetase autoantibodies and AMA received IVIG more often than Ro52-positive/AMA-negative and anti-synthetase-positive/AMA-negative patients, respectively (Supplemental Table 2). The associations with dysphagia and IVIG use within these autoantibody subgroups were modest and did not remain significant when correcting for multiple comparisons. There were no associations with cardiomyopathy or Rituximab use in the autoantibody subgroup analyses (Supplemental Table 2). Additional adjustment by anti-Ro52 autoantibodies of the multivariate analysis did not modify the AMA associations with specific clinical features and increased severity of the disease. We were unable to assess co-positivity to more than one MSA as most patients with complete data were positive for a single MSA.

The presence of AMA in JIIM was not disease specific. However, it is notable that all children with AMA had moderate to severe disease at onset and episodes of falling, and 3/4 had dysphagia and/or dyspnea on exertion without the presence of ILD. None had cardiomyopathy or cardiac involvement.

## Discussion

In this study, we found that AMA are present in 5% of adult myositis patients and 1% of JIIM patients. Importantly, we determined that the prevalence of AMA in IIM is much higher than we previously reported due to more thorough, systematic testing of the Johns Hopkins Myositis Center Longitudinal Cohort [3]. We found that adult myositis patients with AMA more often had chronic muscle weakness, DM-specific rashes, Raynaud’s phenomenon, and dysphagia. In addition, we confirmed that patients with AMA are more likely to have cardiomyopathy, as we hypothesized based on our prior descriptive study of AMA in IIM [3]. Notably, only 2 of our originally reported AMA-positive patients with cardiomyopathy [3] were seen sequentially between 2011 and 2015 and included in our current analysis. Lastly, we report that AMA-positive patients received certain medications more frequently. However, this could be due to the high number of AMA-negative IBM patients who often do not receive such therapies.

Although we did not find an increased frequency of muscle weakness at disease onset in patients with AMA, we observed a higher incidence of muscle weakness throughout disease course. This association parallels our prior report which describes subtle muscle involvement early with subsequent muscle atrophy [3]. We did not, however, observe more necrotizing myopathy in muscle histopathology [3], nor did we observe increased muscle atrophy or MRI findings that would distinguish a pattern of AMA-associated myopathy [11].

In addition to cardiomyopathy, the presence of AMA was associated with Raynaud’s phenomenon, suggesting that these autoantibodies may occur more often in patients with symptoms of vasomotor instability. Although pathogenesis connecting vasomotor instability and cardiomyopathy in myositis patients with AMA is unclear, it is possible that endothelial damage and oxidative stress may play a role. These destructive processes are a known cause and consequence of dysregulated endothelial mitochondria and can even inhibit mitochondrial respiration in the myocardium [12], which may influence the development of heart disease [13]. In fact, prior descriptions of myositis patients with AMA, vasculopathy, and cardiomyopathy detail striking alterations of mitochondria on histochemical examination, suggestive of a mitochondrial myopathy [14]. Although we found AMA to be associated with similar clinical manifestations, we did not find evidence of mitochondrial dysfunction based on muscle histochemistry. Future studies are required to assess the relationship of vasculopathy, cardiomyopathy, and mitochondrial dysfunction in patients who develop AMA. Despite this, our data importantly show that AMA may be biomarkers that heighten clinical suspicion for the development of these disease manifestations.

Lastly, our data suggest that unlike other myositis-associated autoantibodies found in both JIIM and IIM [15], the presence of AMA in children is not disease-specific and thus may not be clinically relevant. Why AMA would be rare in JIIM compared to adults remains unknown.

This study has several limitations. First, data regarding cardiac involvement was collected retrospectively, and many patients did not undergo complete cardiac evaluations; thus, patients with subclinical cardiac pathology could have been missed in our analysis. In addition, 7 patients had cardiomyopathy based on physician documentation alone. Furthermore, clinical data regarding MSA-negative patients was incomplete and thus not included in our analysis. Finally, some patients may have had variable or limited follow-up over time. Due to this and the long-term nature of our cohort, recently developed measures of disease activity and outcomes were not evaluated in many patients.

These limitations notwithstanding our study show that AMA are present in 5% of IIM patients and are associated with chronic weakness, cardiomyopathy, dysphagia, vasomotor instability, and more immunosuppressive therapy. Overall, our data suggest that AMA may be used as biomarkers in disease management and suggest adult patients with AMA warrant a higher index of suspicion for the development of dysphagia and/or cardiomyopathy, which may require modification of therapy.

## Supplementary information

ESM 1 (DOCX 25 kb)

## Data Availability

The authors have full control of all primary data and agree to allow the journal to review the data if requested.

## Code availability

Stata/MP V.14.1
