# Supplementary material for: Anti-mitochondrial autoantibodies are associated with cardiomyopathy, dysphagia, and features of more severe disease in adult-onset myositis
Source: Clin Rheumatol. 2021 Apr 13;40(10):4095–100. doi: 10.1007/s10067-021-05730-7 (PMC8463345; doi:10.1007/s10067-021-05730-7)
Supplement: Supplementary file 1 — (DOCX 25 kb) [file 10067_2021_5730_MOESM1_ESM.docx]

| Supplemental Table 1. Laboratory features^†^ in adult myositis patients with and without AMA | | | | | |
| --- | --- | --- | --- | --- | --- |
|  | **AMA positive % (n/N) or Mean (SD)** | **AMA negative % (n/N) or Mean (SD)** | **Univariate p-value** | **Multivariate**  **p-value** | **Total** |
| Muscle Enzymes | **(N=30)** | **(N=450)** |  | | **(N=480)** |
| Maximum CK | 986 (155-3513) | 1022 (294-4000) | 0.4 | 1.0 | 1011 (281-4000) |
| Maximum aldolase | 20.5 (29.2) | 29.9 (112.5) | 0.7 | 0.8 | 29.3 (109.1) |
|  | | | | | |
| Thigh MRI results | **(N=17)** | **(N=225)** |  | | **(N=242)** |
| Muscle edema | 88% (15) | 85% (192) | 1.0 | 0.3 | 86% (207) |
| Atrophy | 53% (9) | 57% (128) | 0.8 | 0.2 | 57% (137) |
| Fatty replacement | 71% (12) | 73% (165) | 0.8 | 0.4 | 73% (177) |
| Fascial edema | 41% (7) | 50% (112) | 0.5 | 0.3 | 49% (119) |
|  | | | | | |
| Muscle biopsy results | **(N=15)** | **(N=201)** |  | | **(N=216)** |
| Necrotizing myopathy | 33% (5) | 32% (64) | 1.0 | 0.9 | 32% (69) |
| Degenerating fibers | 87% (13) | 89% (176) | 0.7 | 0.9 | 89% (189) |
| Perifascicular atrophy | 33% (5) | 15% (31) | 0.1 | 0.9 | 17% (36) |
| Perivascular inflammation | 47% (7) | 49% (98) | 0.9 | 0.5 | 49% (105) |
| Primary inflammation | 20% (3) | 44% (88) | 0.07 | 0.7 | 42% (91) |
| Cox-negative fibers^a^ | 52% (68) | 45% (5) | 0.7 | 0.9 | 51% (73) |
| *Dichotomous variables were expressed as percentage (count) and continuous variables as mean (SD). Univariate comparisons of continuous variables were made using Student´s t-test while dichotomous variables were compared either using chi-squared test or Fisher´s exact test, as appropriate. Multivariate comparisons were performed using linear regression for continuous variables and logistic regression for dichotomous variables. All multivariate comparisons were adjusted by gender and clinical group (IBM or autoantibody group).*  ^†^Obtained at first clinic visit  ^a^ Cox-negative fibers: > 5 cox-negative fibers per frozen section  Abbreviations: CK: creatinine kinase; MRI: magnetic resonance imaging | | | | | |

| Supplemental Table 2: Frequency of clinical features and medication usage in adult patients with and without AMA within MSA and MAA subgroups | | | | | |
| --- | --- | --- | --- | --- | --- |
|  | **AMA positive** | **AMA negative** | **Univariate**  **P value** | **Multivariate**  **P value** | **Total** |
| Cardiomyopathy |  |  |  |  |  |
| Anti-Ro52 + (n=144) | 11% (2) | 6% (11) | 0.6 | 0.3 | 7% (13) |
| Anti-synthetase + (n=82) | 33% (1) | 5% (3) | 0.2 | 1.0 | 6% (4) |
| Anti-NXP2 + (n=42) | 0% (0) | 0% (0) | . |  | 0% (0) |
| Anti-TIF1+ (n=54) | 14% (1) | 3% (1) | 0.3 |  | 5% (2) |
| Anti-Mi2 + (n=34) | 0% (0) | 0% (0) | . |  | 0% (0) |
| Anti-MDA5 + (n=25) | 0% (0) | 0% (0) | . |  | 0% (0) |
| Anti-SRP + (n=28) | 0% (0) | 7% (2) | 1.0 | . | 7% (2) |
| Anti-HMGCR + (n=62) | 0% (0) | 5% (3) | 1.0 | . | 5% (3) |
|  | | | | | |
| Dysphagia |  |  |  |  |  |
| Anti-Ro52 + (n=144) | 61% (11) | 37% (69) | 0.05 | 0.2 | 39% (80) |
| Anti-synthetase + (n=82) | 67% (2) | 35% (28) | 0.6 | 0.3 | 37% (30) |
| Anti-NXP2 + (n=42) | 80% (4) | 63% (24) | 0.6 | 0.5 | 65% (28) |
| Anti-TIF1+ (n=54) | 57% (4) | 49% (23) | 1.0 | 0.5 | 50% (27) |
| Anti-Mi2 + (n=34) | 100% (3) | 45% (14) | 0.2 | . | 50% (17) |
| Anti-MDA5 + (n=25) | 33% (1) | 36% (8) | 1.0 | 1.0 | 36% (9) |
| Anti-SRP + (n=28) | 100% (1) | 52% (14) | 1.0 | . | 54% (15) |
| Anti-HMGCR + (n=62) | 100% (3) | 35% (21) | 0.05 | . | 38% (24) |
|  | | | | | |
| IVIG |  |  |  |  |  |
| Anti-Ro52 + (n=144) | 55% (12) | 30% (62) | 0.02 | 0.1 | 33% (74) |
| Anti-synthetase + (n=82) | 100% (3) | 29% (23) | 0.03 | . | 32% (26) |
| Anti-NXP2 + (n=42) | 40% (2) | 45% (17) | 1.0 | 0.9 | 44% (19) |
| Anti-TIF1+ (n=54) | 71% (5) | 57% (27) | 0.7 | 0.6 | 59% (32) |
| Anti-Mi2 + (n=34) | 67% (2) | 52% (16) | 1.0 | 0.8 | 53% (18) |
| Anti-MDA5 + (n=25) | 67% (2) | 27% (6) | 0.2 | 0.2 | 32% (8) |
| Anti-SRP + (n=28) | 100% (1) | 33% (9) | 0.4 | . | 36% (10) |
| Anti-HMGCR + (n=62) | 67% (2) | 43% (26) | 0.6 | 0.6 | 44% (28) |
|  | | | | | |
| Rituximab |  |  |  |  |  |
| Anti-Ro52 + (n=144) | 27% (6) | 15% (30) | 0.1 | 0.09 | 16% (36) |
| Anti-synthetase + (n=82) | 67% (2) | 20% (16) | 0.1 | 0.07 | 22% (18) |
| Anti-NXP2 + (n=42) | 20% (1) | 13% (5) | 0.5 | 0.7 | 14% (6) |
| Anti-TIF1+ (n=54) | 29% (2) | 11% (5) | 0.2 | 0.2 | 13% (7) |
| Anti-Mi2 + (n=34) | 33% (1) | 26% (8) | 1.0 | 1.0 | 26% (9) |
| Anti-MDA5 + (n=25) | 67% (2) | 14% (3) | 0.09 | 0.1 | 20% (5) |
| Anti-SRP + (n=28) | 0% (0) | 63% (17) | 0.4 | . | 61% (17) |
| Anti-HMGCR + (n=62) | 0% (0) | 15% (9) | 1.0 | . | 14% (9) |
| *Dichotomous variables were expressed as percentage (count) and compared either using chi-squared test or Fisher´s exact test, as appropriate. Multivariate comparisons were performed using logistic regression for dichotomous variables. All multivariate comparisons were adjusted by gender and clinical group (IBM or autoantibody group).*  Abbreviations: AMA: anti-mitochondrial autoantibodies, TIF1: transcription intermediary factor 1, NXP2: nuclear matrix protein-2, MDA5: melanoma differentiation associated protein-5, SRP: signal recognition particle, HMGCR: 3-Hydroxy-3-Methylglutaryl-CoA Reductase | | | | | |
